# Supplementary material for: Targeted Chromosomal Barcoding Establishes Direct Genotype-Phenotype Associations for Antibiotic Resistance in Mycobacterium abscessus
Source: Microbiol Spectr. 2023 Mar 29;11(3):e05344-22. doi: 10.1128/spectrum.05344-22 (PMC10269753; doi:10.1128/spectrum.05344-22)
Supplement: Supplemental file 1 — Fig. S1 to S5. Download spectrum.05344-22-s0001.pdf, PDF file, 8.3 MB [file spectrum.05344-22-s0001.pdf]

SUPPLEMENTARY FIGURES

Targeted chromosomal barcoding establishes direct genotype-phenotype associations for antibiotic resistance in *Mycobacterium abscessus*

Juan Calvet-Seral, Estefanía Crespo-Yuste, Vanessa Mathys, Hector Rodriguez-Villalobos, Pieter-Jan Ceyssens, Anandi Martin, Jesús Gonzalo-Asensio

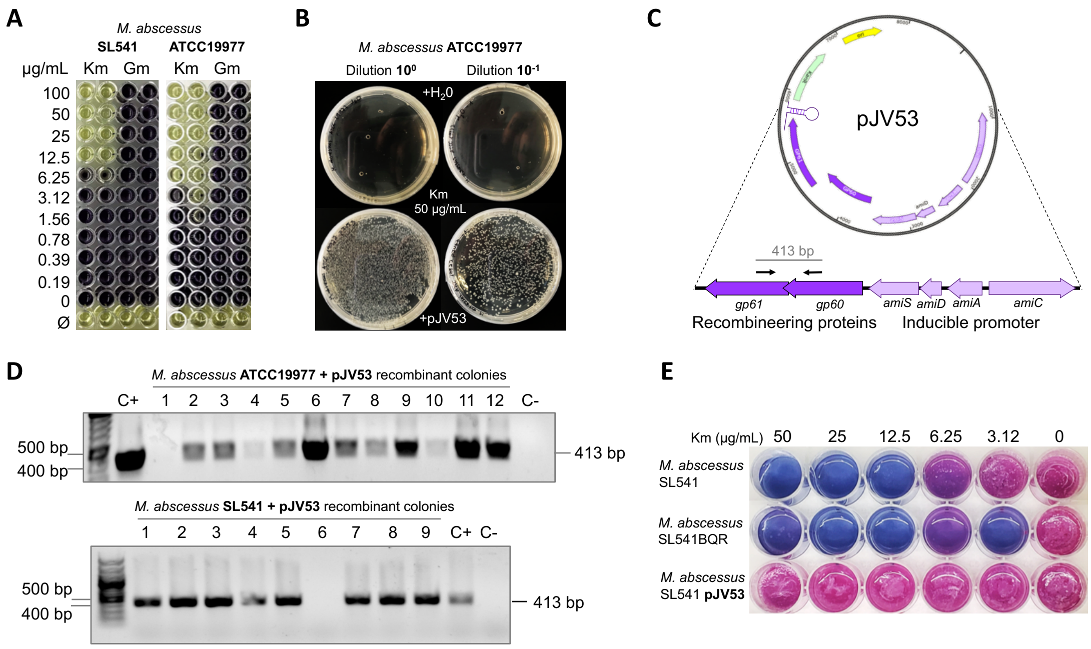

Supplementary Figure S1: (**A**) MTT in 7H9Tyl-ADC to determine the MIC of clinical isolate SL541 and the laboratory reference strain ATCC19977 against kanamycin (Km), and gentamycin (Gm) as positive control. Wells with viable bacteria are shown in dark purple, whereas wells with absence of growth are shown in yellow. Note the susceptibility against kanamycin in both strains. (**B**) Selection of Km resistant colonies after electroporation with pJV53 plasmid. Note the absence of bacterial growth in non-transformed bacteria, which minimizes the chance of recovering spontaneous mutant against this antibiotic. (**C**) Graphical representation of the pJV53 plasmid used to electroporate *M. abscessus* cells in order to obtain transformants carrying a recombineering system. The scheme shows positions of gp60 Fw and gp61 Rv oligonucleotides used to specifically confirm the presence of the plasmid. (**D**) Colony PCR with gp60 FW and gp61 Rv oligonucleotides of different *M. abscessus* ATCC19977 and SL541 transformants to confirm the presence of pJV53 plasmid. (**E**) REMA assay in 7H10-ADC agar to determine the MIC against Km of *M. abscessus* SL541-derived strains. Wells with viable bacteria are shown in pink, whereas wells with absence of growth are shown in blue, after addition of resazurin.

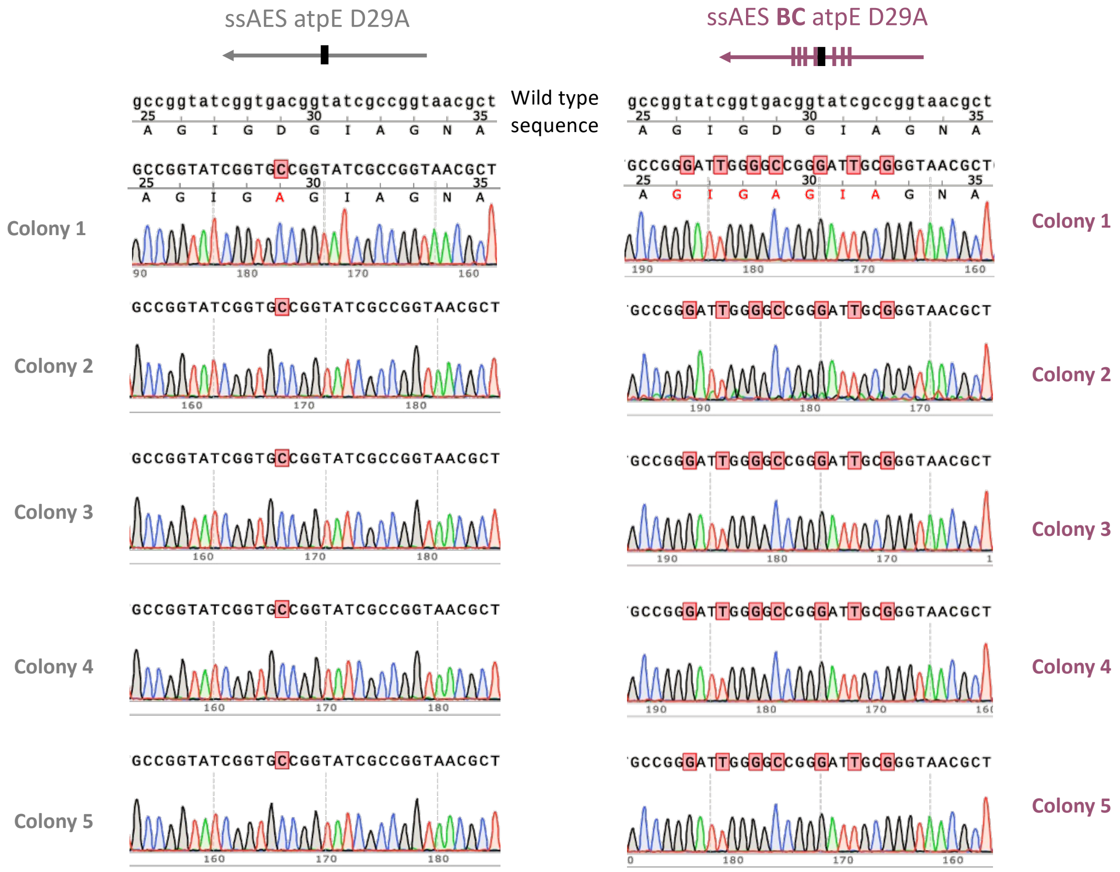

Supplementary Figure S2. Sanger sequencing chromatograms of all recombinant colonies recovered of *M. abscessus* SL541 electroporated with “ssAES atpE **D29A**” (left) and “ssAES **BC** atpE **D29A**” (right).

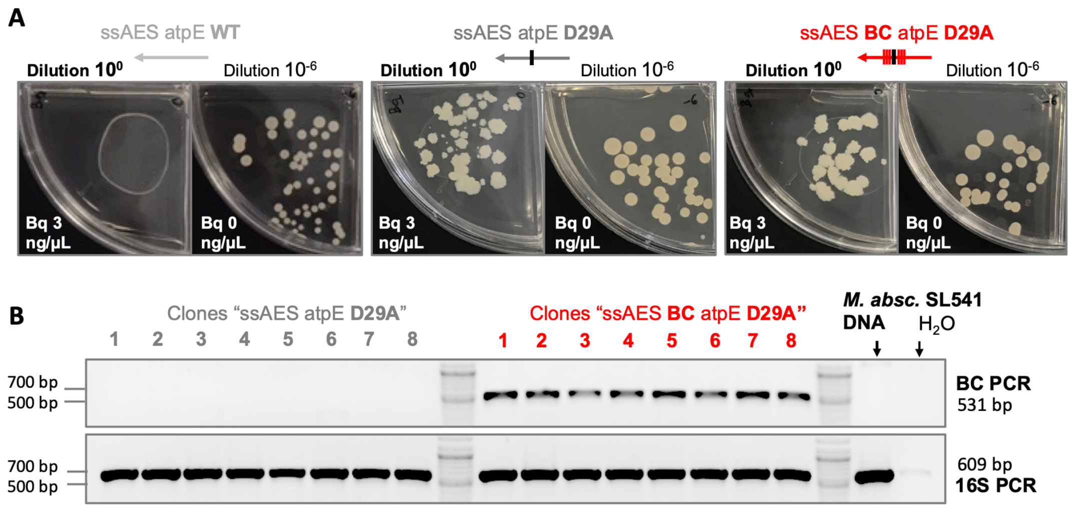

Supplementary Figure S3. (**A**) *M. abscessus* ATCC19977 carrying pJV53 was transformed with "ssAES *atpE* WT", "ssAES *atpE* D29A" and "ssAES BC *atpE* D29A" and subsequently plated in absence (dilution 10^{-6}) or presence (dilution 10^{0}) of bedaquiline at 3 µg/mL. (**B**) Bedaquiline resistant colonies were subjected to specific Barcode-PCR. The presence of a 531 bp band is indicative of the chromosomal replacement of the *atpE* allele with the barcode AES bearing the Bedaquiline resistant D29A mutation. Amplification of a 609 bp band corresponding to the 16S housekeeping gene is also shown as positive control for amplification.

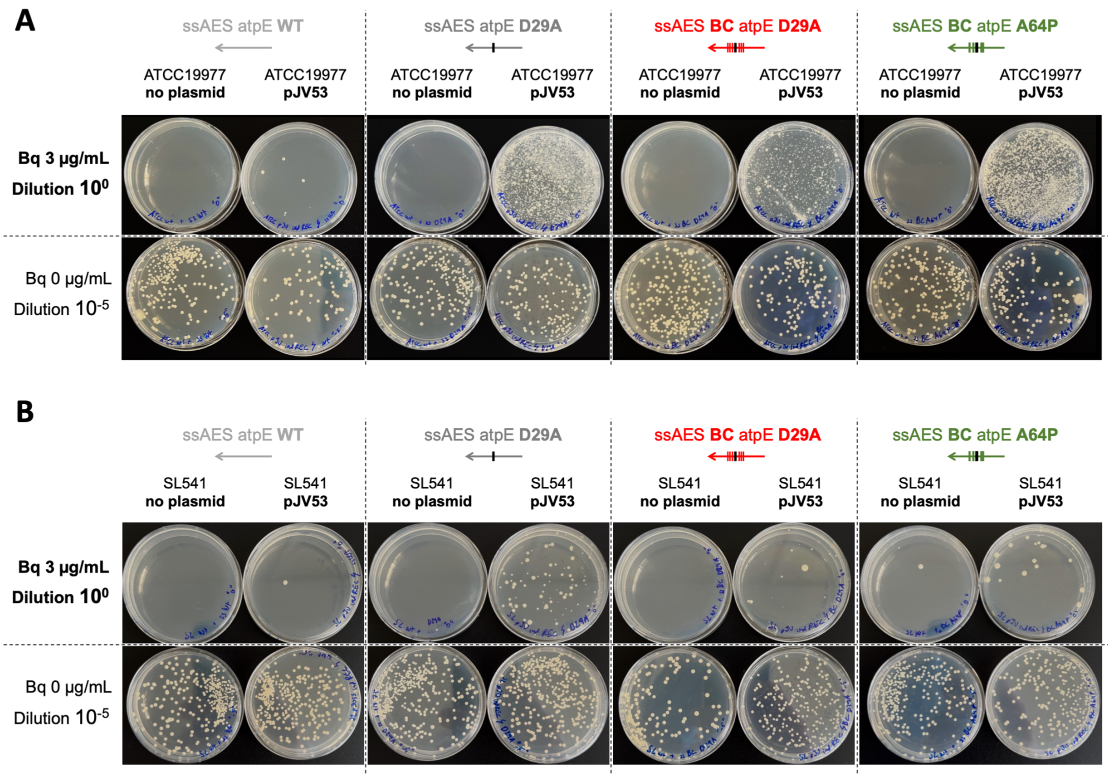

Supplementary Figure S4: (**A and B**) *M. abscessus* ATCC19977 (**A**) and SL541 (**B**) with (right panels) or without pJV53 (left panels) transformed with "ssAES *atpE* WT", "ssAES *atpE* D29A", "ssAES BC *atpE* D29A" and "ssAES BC *atpE* A64P". Transformants were plated in presence (dilution 10^0) or absence (dilution 10^{-5}) of Bedaquiline 3 µg/mL. Note the absence of bacterial growth in wild type bacteria not transformed with pJV53. In contrast, pJV53-transformed bacteria resulted in increased CFU numbers, which were significantly higher in transformants with AES conferring bedaquiline resistance ("ssAES *atpE* D29A", "ssAES BC *atpE* D29A" and "ssAES BC *atpE* A64P"), when compared with the wild type *atpE* AES. Differences in CFU numbers are not related to differences in bacteria plated as observed in the CFU grown in the absence of Bedaquiline.

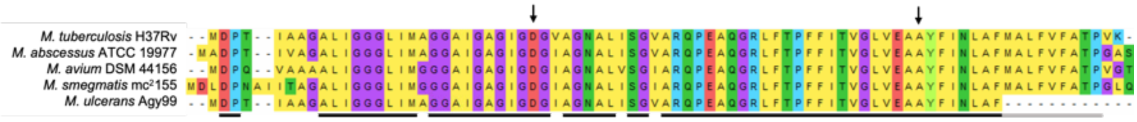

Supplementary Figure S5. Alignment by MUSCLE algorithm in MEGA software of *atpE* encoded protein (ATP synthase subunit C) of different mycobacteria. Residues mutated in this study are marked with arrows. 100% identity regions are underlined, showing high conservation of the protein (black line, conserved in the five species compared, grey line, conserved in all but deleted in *M. ulcerans* Agy99 strain).
